# Assessment of Toxic Effects of Ochratoxin A in Human Embryonic Stem Cells

**DOI:** 10.3390/toxins11040217

**Published:** 2019-04-10

**Authors:** Slaven Erceg, Eva María Mateo, Iván Zipancic, Francisco Javier Rodríguez Jiménez, María Amparo Pérez Aragó, Misericordia Jiménez, José Miguel Soria, Mª Ángeles Garcia-Esparza

**Affiliations:** 1 Stem Cells Therapies in Neurodegenerative Diseases Lab, Research Center “Principe Felipe”, Valencia 46012, Spain; frodriguez@cipf.es (F.J.R.J.); mparago@cipf.es (M.A.P.A.); 2 Department of Microbiology and Ecology, University of Valencia, Valencia 46100, Spain; eva.mateo@uv.es (E.M.M.); misericordia.jimenez@uv.es (M.J.); 3 Department of Biomedical Sciences, School of Health Sciences, Universidad Cardenal Herrera-CEU, CEU Universities, Valencia 46115, Spain; ivan.zipancic@uchceu.es; 4 Department of Biomedical Sciences, School of Health Sciences, Universidad Cardenal Herrera-CEU, CEU Universities, Elche, Alicante 03204, Spain; Jose.soria@uchceu.es; 5 Department of Pharmacy, School of Health Sciences, Universidad Cardenal Herrera-CEU, CEU Universities, Elche, Alicante 03204, Spain; Maria.garcia2@uchceu.es

**Keywords:** Ochratoxin A (OTA), human Stem Cells, mycotoxins, cells, cytotoxicity, cell culture

## Abstract

Ochratoxin A (OTA) is a mycotoxin produced by different *Aspergillus* and *Penicillium* species, and it is considered a common contaminant in food and animal feed worldwide. On the other hand, human embryonic stem cells (hESCs) have been suggested as a valuable model for evaluating drug embryotoxicity. In this study, we have evaluated potentially toxic effects of OTA in hESCs. By using in vitro culture techniques, specific cellular markers, and molecular biology procedures, we found that OTA produces mild cytotoxic effects in hESCs by inhibiting cell attachment, survival, and proliferation in a dose-dependent manner. Thus, we suggest that hESCs provide a valuable human and cellular model for toxicological studies regarding preimplantation stage of human fetal development.

## 1. Introduction

Human pluripotent stem cells (hPSCs) represent heterogeneous populations, including induced pluripotent stem cells (iPSCs), endogenous plastic somatic cells, and embryonic stem cells (ESCs). Human ESCs (hESCs) are derived from the inner cell mass of the blastocyst, characterized by the ability to self-renew indefinitely and to give rise to all cell types of embryonic lineage (pluripotency) under the guidance of the appropriate chemical, mechanical, and environmental cues [1].

There are high expectations regarding the use of hESCs for treating injuries and degenerative diseases, for modelling complex illnesses and developments, for screening and testing of pharmacological products, and for examining toxicity, mutagenicity, teratogenicity, and potential carcinogenic effects of a variety of environmental factors, including mycotoxins [2,3].

Ochratoxin A (OTA) is the most abundant and toxic member of the ochratoxins, a group of secondary metabolites produced by fungi belonging to the genera *Aspergillus* and *Penicillium* [4,5,6,7]. 

OTA can contaminate a wide variety of foods because of fungal infection in crops, in fields during growth, at harvest, or during storage and shipment. Besides cereals and cereal products, OTA is also found in a range of other food commodities, including coffee, cocoa, wine, beer, pulses, spices, dried fruits, grape juice, pig kidney, and other meat and meat products from non-ruminant animals exposed to foodstuffs contaminated with this mycotoxin [8].

Research into the toxicity of this mycotoxin is mostly centered on its teratogenic [9], nephrotoxic [10], immunotoxic [10], neurotoxic [11,12,13], and carcinogenic [14] effects that result from exposure to a range of different food types, particularly of plant origin, that may be contaminated by OTA [15,16]. The kidney has been considered as the key target organ of OTA toxicity in most of the mammalian species [17]. Additionally, in humans OTA has been found in blood plasma [5,18], and frequent exposure to OTA is attributed to its nephrotoxic effects, especially in children [19]. Several studies have highlighted OTA as a possible causative agent of Balkan endemic nephropathy, an endemic, severe, progressive, and fatal kidney disease found in the Balkan countries [14,19,20]. 

Furthermore, investigations in animal models showed OTA as a neurotoxic agent [21,22]. In addition, different studies in vitro have demonstrated a direct relationship between some environmental products and prenatal development [23]. Thus, although OTA appears to exert multiple biological actions, and is cytotoxic, few studies conducted to date have explored whether OTA negatively affects embryonic development [24,25]. 

During normal embryogenesis, the process of apoptosis removes abnormal or redundant cells from pre-implantation embryos [26]. Induction of apoptosis during early stages of embryogenesis (i.e., following exposure to a teratogen) compromises embryonic development [27,28]. The main methods to study teratogens are either through epidemiological studies in human populations or by controlled exposure in animal models. Previous studies found that OTA induced apoptosis in mammalian cells, including monkey and human kidney epithelial cells, porcine kidney PK15 cells, and human OK cells [29,30,31]. Although these methods are still essential, more reliable and indicative human-based toxicity tests are needed to represent toxicity effects in humans. Due to the ethical issues regarding teratogenic effect assessment of OTA in human embryos, in this study we have used hESCs as an in vitro model for teratogen screening in a human developmental setting using physiologically relevant doses. There is clear evidence that hESCs represent faithful in vitro toxicity models, as a wide range of chemicals were tested and showed adverse effects in these cells [32,33,34,35] with no toxicity in animal models, such as in the case of thalidomide [36]. As hESCs are cells derived from the blastocyst stage, toxicity assays with hESCs can provide toxicity information at a very early stage after fertilization. Having unique proliferation and differentiation capacities toward a wide range of cells in the human body, hESCs closely mimic human embryogenesis [37], thus they offer a unique cellular, developmental, functional, and reproductive human in vitro model for toxicological testing.

The purpose of this study was to assess and determine toxicity of OTA using hESCs as a model for preimplantation embryos. Our data show that (1) hESCs can be used to measure toxicity of food contaminants such as OTA, and (2) OTA exerts its effect through possible mechanisms of apoptosis and oxidative stress.

## 2. Results

### 2.1. Ochratoxin A Reduces the Viability and Decreases the Cellular Proliferation of Human Embryonic Stem Cells (hESCs)

**Figure 1:**
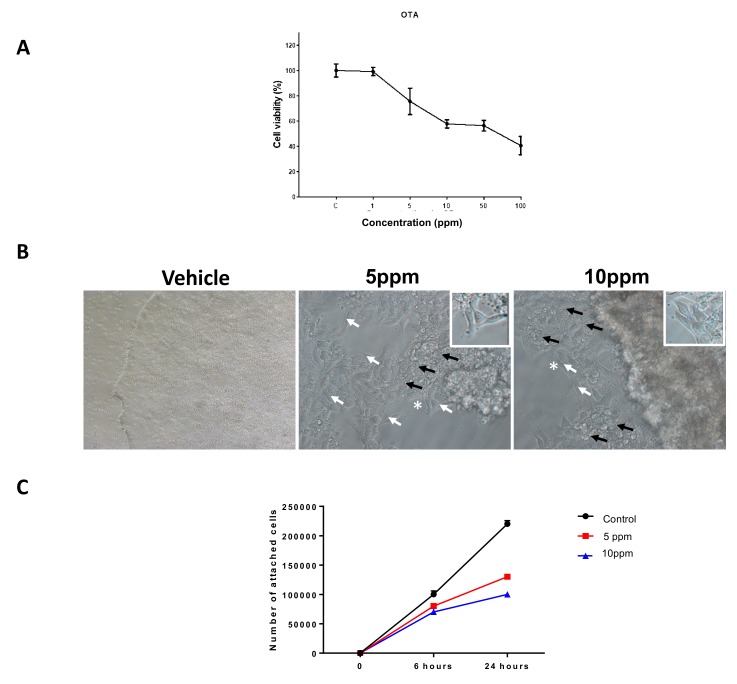
(**A**) Dose-dependent survival rate (MTS assay) of human embryonic stem cells (hESC) at 8 h shows decrease of cell survival to 60% at doses of 10 ppm (*n* = 6). (**B**) Representative bright field micrographs of hESC colonies treated with vehicle (ethanol) or OTA (5 and 10 ppm). White arrows indicate surviving cells and black arrows indicate dead cells. Micrographs show the suitable aspect and shape of surviving cells. White asterisks indicate the area from which the photographs were taken. (**C**) Number of attached cells after 6 and 24 h in two experimental groups compared to control. Magnification times: 20×.

OTA treatment (1–100 ppm) reduced the viability of hESCs in a dose-dependent manner. Evident toxic effects of OTA were observed after 8 h when approximately 60% of cells survived at a concentration of 10 ppm. Similar effects were observed with a concentration of 50 ppm of OTA, and this was considered the 50% effective concentration (EC_50_) (Figure 1A,B). In all treatments, the percentage of colonies that underwent shrinkage during exposure exponentially increased (data not shown).

Since the major OTA effects on cell death were observed with concentrations of 5 and 10 ppm, further experiments were performed using these concentrations. Thus, to determine how OTA solutions affected proliferation of hESCs, the surviving cells after 5 and 10 ppm treatments were detached, seeded, and used in subsequent experiments to assess cell attachment and growth during the following 24 h (Figure 1C). Throughout this process, video data of colonies in each group were collected using the IncuCyte System. All videos were first analyzed to determine whether colonies grew, shrunk, or died during incubation. During the 24 h, an evident decrease of cell growth and attachment was observed in comparison with non-treated cells (Figure 1C).

**Figure 2:**
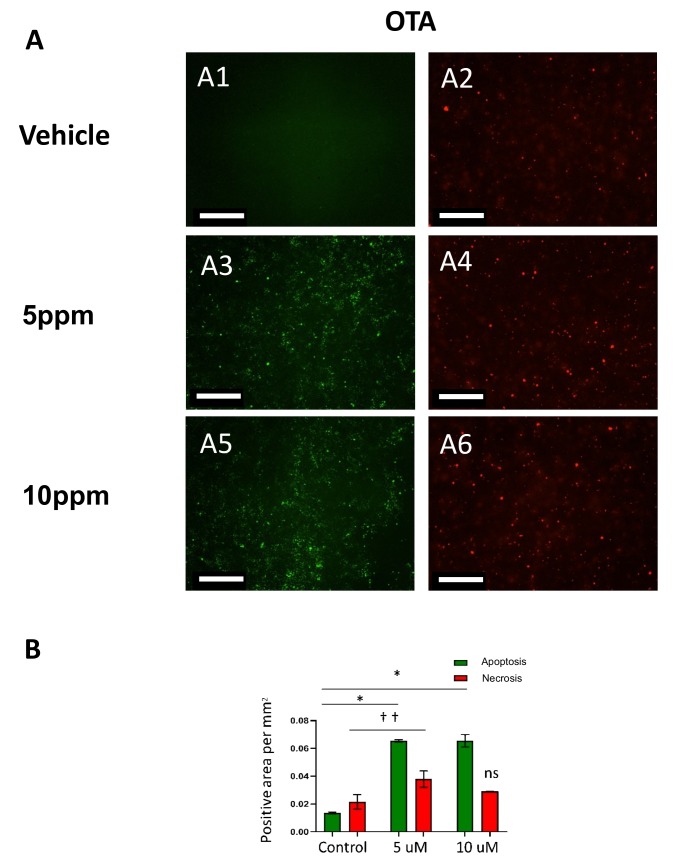
Ochratoxin A (OTA) increases necrotic cells and apoptosis in hESCs at 5 and 10 ppm. (**A**) Representative fluorescent micrographs of hESC colonies treated with vehicle (ethanol in A1, A2), OTA (5 ppm in A3, A4), and (10 ppm in A5, A6) captured after 8 h with the IncuCyte ZOOM. IncuCyte® Cytotox Red Reagent was used for counting necrotic cells (red) and caspase-mediated apoptosis using a kinetic caspase 3/7 reagent (Essen Bioscience) (green). (**B**) The number of “objects” per well was calculated using IncuCyte software and graphed, showing a significant increase in the number of cells undergoing necrosis (red) or caspase-mediated apoptosis when treated with OTA (green) compared to ethanol. (*n* = 3; *, † = *p* ≤ 0.05). Scale bar: 300 µm. Magnification times: 20×.

In order to investigate the role of OTA on cell death and apoptotic processes, the cells were stained with nucleic acid IncuCyte® Cytotox Red Reagent for counting necrotic cells, which was able to penetrate and dye compromised cell membranes associated with dead or dying cells, followed by imaging with the IncuCyte ZOOM every 4 h over a 24 h period. A dose-dependent increase in cell death when treated with OTA was observed across all hESCs (Figure 2A, (A1, A3 and A5) and Figure 2B. The same results regarding cell death were observed over 24 h.

### 2.2. Ochratoxin A (OTA) Induces Caspase-Mediated Apoptosis in hESCs

In addition to cytotoxicity quantification, hESCs were also stained and imaged for caspase- mediated apoptosis using a kinetic caspase 3/7 reagent (Essen BioScience, 300 West Morgan Road, Ann Arbor, MI, USA). The number of “caspase-3 objects” per well was calculated using IncuCyte integrated analysis software and graphed, showing a significant increase in the number of cells undergoing caspase-mediated apoptosis when treated with OTA compared to vehicle treated cells (Figure 2A, (A2, A4 and A6) and Figure 2B). The trend in caspase 3/7 activation in hESCs correlated with their EC_50_ value. 

### 2.3. OTA Increases the Expression of Oxygen Stress Markers in hESCs

**Figure 3:**
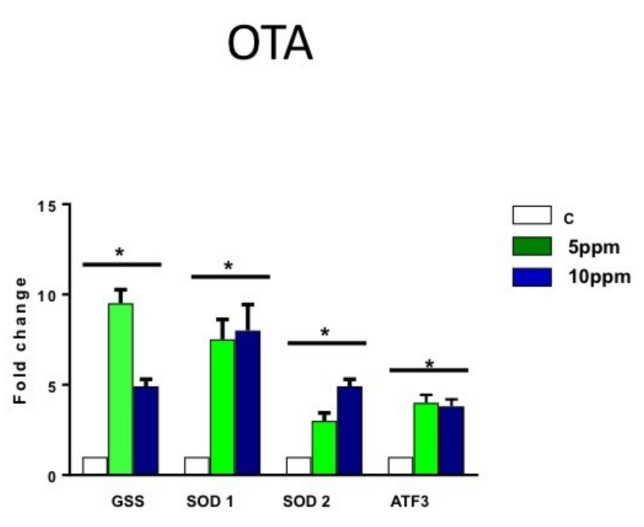
OTA significantly increases the expression of oxygen stress markers in hESCs. Analysis of reactive oxygen stress markers showed a dose-dependent increase of the expression of glutathione synthetase (GSS), superoxide dismutases 1 (SOD1), superoxide dismutases 1 (SOD2), and activating transcription factor 3 (ATF3) as main markers involved in oxidative stress. (*n* = 3; * = *p* ≤ 0.05).

After apoptosis and cytotoxicity assays, the cells were collected, and RT-PCR for main oxidative markers was performed to determine the role of oxidative stress in OTA cytotoxicity. In cells treated with OTA, analysis of reactive oxygen stress markers showed a significant but not dose-dependent increase of the expression of glutathione synthetase (gss), superoxide dismutases 1 (sod1), superoxide dismutases 2 (sod2) and activating transcription factor 3 (atf3) in reactive oxygen species (ROS) for both doses: 5 and 10 ppm (Figure 3). A greater fold-change compared to vehicle control was observed in hESCs, strongly suggesting OTA-induced cell death.

## 3. Discussion

OTA exposure studies have been developed on different cell lines of human and animal models, especially describing the mechanisms associated with increased levels of oxidative stress, DNA, and lipid and protein damage [38]. Embryos are generally more sensitive to chemicals than adults are, and for this reason it is essential to develop faithful human cell assays for preimplantation stages of human development when possible [39]. OTA is a mycotoxin commonly found in food, which can produce serious toxic effects in the organism and, specifically, in the developing brain [40,41]. In this study, we evaluated the impact of OTA exposure in hESCs as a model for pre- and post-implantation of human embryos. OTA showed toxic, dose-dependent effects only after 4 h of treatment. The mechanism through which OTA induces toxicity in vitro is mainly attributed to multiple effects on various subcellular structures, such as loss of membrane integrity, confirmed by LDH leakage assay in other cells [42,43]. In the present study, OTA exhibited cell toxicity via cell mortality, confirmed by MTS, and through mechanisms of apoptosis and oxidative stress. Our results are in line with earlier studies, which have demonstrated that OTA-induced oxidative stress leads to cytotoxicity and apoptosis in Neuro-2a cells [43], highlighting that elevated ROS is a principle event in oxidative stress in cells treated with OTA. Our results corroborate other findings where OTA was confirmed as a potent ROS inducer [44,45,46]. Results of our study suggest that OTA cytotoxicity is mediated by oxidative stress in a dose-dependent manner. Although the oxidative species were not measured, the significant increase of expression of main oxidative stress markers, such as GSS, SOD 1, SOD2, and ATF3, strongly indicate that oxidative stress is one of the underlying mechanisms for OTA-induced loss of cell viability and DNA damage. Since mitochondria events are the major generator of ROS, mitochondria could play a crucial role in toxicity of OTA [47]. Generation of free radicals and other oxidative species triggers lipid peroxidation and permeability of the mitochondrial membrane, which produces apoptotic cell death [47,48]. Indeed, it was previously shown that OTA treatment leads to loss of mitochondrial membrane potential and DNA damage in a dose-dependent manner [43]. Our study confirms results obtained by Sava et al. [11,22], in which the authors tested neural stem/progenitor cells (NSCs) prepared from the hippocampus of an adult mouse brain for their vulnerability to OTA in vitro. In that study the authors observed that OTA caused a dose- and time-dependent decrease in viability of both proliferating and differentiating NSCs. Along with decreased viability, OTA elicited pronounced oxidative stress, evidenced by a robust increase in total and mitochondrial SOD activity. This study concluded that greater vulnerability to the toxin exhibited proliferating number of NSCs compared to differentiated, more mature neurons, despite robust DNA repair and antioxidant responses. Further studies need to be performed in order to clarify whether the same mechanisms of oxidative stress are triggered in hESC by OTA. Our results are in line with previous studies, which have reported OTA as a trigger for the caspase-9 and caspase-3 activation with potential mitochondrial membrane loss in different human primary cells [49,50,51]. To our knowledge, the present study is the first one describing the effects of OTA in a prenatal human cellular model, demonstrating the importance of assessing toxicity in early stages of development. In this context, it is crucial to develop new simple and faithful in vitro assays that are able to screen the effects of environmental and food chemicals in various stages of the developing fetus. To this purpose, many assays, such as explants of rodent embryos [52,53] or embryonic bodies derived from mESC to model post-implantation development, have been developed [54]. The approach used in this study represents a quick and simple human in vitro method for assessing environmental toxicants in hESCs, a model for the inner cell mass of preimplantation embryos already used for other environmental pollutants [55] such as tobacco smoke [35] or thalidomide [56]. This study may contribute to elucidating the mechanisms underlying OTA teratogenicity in the early days of human fetal development.

## 4. Materials and Methods

### 4.1. Undifferentiated hESC Line Maintenance 

WA09 hESCs were obtained from the WiCell Research Institute (Madison, WI, USA) and were maintained in feeder-free conditions using mTeSR1 media (StemCell Technologies, Vancouver, BC, Canada) on hESC-qualified Matrigel (BD Biosciences, San Jose, CA, USA) coated plates. To maintain the undifferentiated stem cell population, differentiated colonies were removed daily through aspiration and medium was replaced. Additionally, the hESCs were only used in experiments up to passage 40 and were karyotyped approximately every 10 passages to minimize and monitor the potential for genetic instability. hESCs were passaged at 90%–95% confluence (approximately every 7 days) using Accutase. Cell cultures were maintained at 37 °C under 5% CO_2_.

### 4.2. In vitro Culture of hESCs

All experimental treatments were carried out in 96-well plates coated with Matrigel. To minimize plating variability and increase reproducibility, hESCs were removed from a 6-well plate using TrypLE (Life Technologies). The cells were washed with DMEM/F12 (Dulbecco´s Modified Eagle Medium F-12 Nutrient Mixture (Ham), GIBCO, Paisley, Scotland, UK) and re-suspended in mTeSR1 that contained 10 uM/L Y27632 Rho-associated kinase inhibitor (Merck KGaA/Calbiochem, Darmstadt, Germany). The rho-associated kinase inhibitor was added to the plating media to increase plating efficiency by decreasing dissociation-induced apoptosis. Five thousand hESCs were plated as a single cell suspension and maintained in an undifferentiated state until 80% confluence was reached.

### 4.3. Analysis of Cellular Viability

Analysis of cellular viability in the presence of respiratory inhibitors was performed using MTS assay (Promega, G1111, Promega Corporation, Madison, WI, USA) according to the manufacturer’s instructions. Briefly, hESCs were seeded at a density of 5000 cells per well in Matrigel-treated 96-well culture microplates in 100 μL of culture media, and they were incubated for 4 h at 37 °C. The cells were used when 80% confluence was reached. After 24 h of compound (ethanol) treatment, 20 μL of MTS reagent was applied to each well of a 96-well plate. Absorbance at 490 nm was recorded after 2 h incubation. Sextuplets were prepared for each condition.

### 4.4. hESC Compound Exposures

hESCs were treated with OTA at different concentrations equivalent to previous and published in vitro studies [21]. To test OTA exposure, all compound stock solutions were made with ethanol.

### 4.5. Toxin Preparation

A standard of OTA was supplied by Sigma-Aldrich (Alcobendas, Spain). The OTA standard was dissolved in ethanol (Ethanol HPLC 99.5%-gradient grade, Burker, Deventer, Netherlands) to give a stock solution of 1000 µg/mL (ppm). OTA solutions of 100, 50, 10, 5, and 1 ppm were prepared by dilution of suitable aliquots of the stock solution with ethanol. An aliquot of these mycotoxin solutions was added to the wells containing the cell cultures to obtain the final concentration of the toxin. Blank controls having no mycotoxin, but the same volume of solvent, were performed in parallel.

### 4.6. RNA Extraction and Reverse Transcriptase Polymerase Chain Reaction (RT-PCR and qRT-PCR) 

Cells were collected by centrifugation, and total RNA was isolated with the RNeasy Mini Kit (Qiagen, Hilden, Germany) following the manufacturer´s instructions. They were treated with DNase1 to remove any genomic DNA contamination. QuantiTect Reverse Transcription Kit (Qiagen) was used to carry out cDNA synthesis from 1 μg of total RNA according to the manufacturer’s instructions. For quantitative real-time PCR (qRT-PCR), the relative quantification analysis was performed using a CFX96 RealTime PCR Detection system and C1000 Thermal Cycler (Bio-Rad, Hercules, CA, USA). The PCR cycling program consisted of denaturing at 95 °C for 10 min followed by 40 cycles of 95 °C for 15 s and annealing/elongation at 60 °C for 1 min. The reactions were done in triplicate using TaqMan Gene Expression Master Mix and the following TaqMan probes (Applied Biosystems, Foster City, CA, USA): SOD1 (Hs00533490_mL), SOD2 (Hs00167309_mL), GSS (Hs00609286_mL), and ATF3 (Hs00231069_mL). PCR was done in triplicate, and the expression of polymerase 2A (POL2A; Hs00172187_mL) was used as three endogenous controls to normalize the variations in cDNA quantities from different samples. The results were analyzed using Bio-Rad CFX software (CFX Maestro Software for Bio-Rad CFX Real-Time PCR Systems) and Microsoft Excel (software version 16.16.8 (190312), 2018, Microsoft, Redmond, WA, USA). 

### 4.7. Cytotoxicity and Apoptosis Assays

IncuCyte ZOOM Live-Cell Imaging system (Essen Bioscience, Ann Arbor, MI, USA) was used for kinetic monitoring of cytotoxicity and apoptotic activity of OTA in hESCs. These two assays were performed at the same time. Five thousand hESC cells were seeded at day 3 in mTESR medium in each of the 96-well plates, in such manner that by day 1 the cell confluence was approximately 30%. Cells were treated with increasing two concentrations (5 and 10 ppm) of OTA in the presence of 5 μM of Caspase 3/7 Apoptosis Assay Reagent (Essen Bioscience). The Caspase 3/7 reagent labeled apoptotic cells yielding green fluorescence. At the same time, IncuCyte® Cytotox Red Reagent for counting dead cells was applied. This reagent labeled dead cells yielding red fluorescence. The plate was scanned, and fluorescent and phase-contrast images were acquired in real time every 4 h from 0 to 48 hours post treatment. Normalized green object count per well at each time point and quantified time-lapse curves were generated by IncuCyte ZOOM software (IncuCyte® ZOOM Live-Cell Analysis Systems, 2018. Essen BioScience, 300 West Morgan Road, Ann Arbor, MI, USA). Ratios of caspase 3/7 level in OTA-treated cells compared to vehicle were plotted in Microsoft Excel. The cells were monitored for confluence. At a confluence of 50% we performed the experiment, monitoring cell growth using the IncuCyte System to capture phase contrast images every 2 h, and analyzed results using the integrated confluence algorithm. Caspase 3/7 diluted reagent at 1:1000 (5 µM final concentration) and Cytotox Red Reagent (final volume of 50 µL/well) or vehicle (ethanol) were added to the wells. Then, the medium was aspirated. The images were captured every 2–3 h (10× or 20×) in the IncuCyte® System.

### 4.8. Statistical Analysis 

Statistical analyses of qRT-PCR data from at least three biological replicates were calculated using Student’s t-test using GraphPad Prism 5.02 (GraphPad Software 2365 Northside 560 San Diego, CA, USA).
